# Functional Characterization of *Cucumis metuliferus* Proteinase Inhibitor Gene (*CmSPI*) in Potyviruses Resistance

**DOI:** 10.3390/v7072799

**Published:** 2015-07-09

**Authors:** Chia-Wei Lin, Mei-Hsiu Su, Yu-Tsung Lin, Chien-Hung Chung, Hsin-Mei Ku

**Affiliations:** Agronomy Department National Chung Hsing University, 250 Kuo Kuang Road, Taichung 402, Taiwan; E-Mails: kawey1224@hotmail.com (C.-W.L.); mika740428@hotmail.com (M.-H.S.); pqf292@gmail.com (Y.-T.L.); chienhun@hotmail.com (C.-H.C.)

**Keywords:** Proteinase inhibitors (PIs), *Cucumis metuliferus*, *Papaya ringspot virus* (PRSV), RNA interference (RNAi), *Potato virus Y* (PVY)

## Abstract

Proteinase inhibitors are ubiquitous proteins that block the active center or interact allosterically with proteinases and are involved in plant physiological processes and defense responses to biotic and abiotic stresses. The *CmSPI* gene identified from *Cucumis metuliferus* encodes a serine type PI (8 kDa) that belongs to potato I type family. To evaluate the effect of silencing *CmSPI* gene on *Papaya ringspot virus* resistance, RNA interference (RNAi) with an inter-space hairpin RNA (ihpRNA) construct was introduced into a PRSV-resistant *C. metuliferus* line. *CmSPI* was down-regulated in *CmSPI* RNAi transgenic lines in which synchronously PRSV symptoms were evident at 21 day post inoculation. Alternatively, heterogeneous expression of *CmSPI* in *Nicotiana* *benthamiana* was also conducted and showed that *CmSPI* can provide resistance to *Potato virus Y*, another member of *Potyvirus*, in transgenic *N. benthamiana* lines. This study demonstrated that *CmSPI* plays an important role in resistant function against potyviruses in *C. metuliferus* and *N. benthamiana*.

## 1. Introduction

Proteinase inhibitors (PIs) are commonly found in the plant kingdom and have been identified mainly in plant shoots and storage tissues [1]. At least 74 PI families have been reported based on structural homology, topological relationships, and relative site [2,3,4]. Plant PIs (PPIs) are involved in flowering, seed germination, protein storage, and programmed cell death. PPIs are also induced in plants in responses to wounding and pathogen infections [5]. Several studies have showed that PPIs are effective against predators or pathogens such as insects, nematodes, fungal, and viruses [6,7]. For example, introduction and expression of soybean Kuntiz trypsin inhibitor and Bowman-Birk inhibitor in sugarcane can confer resistance to sugarcane borer *Diatraea saccharalis* [8]. In *Arabidopsis*, overexpression of rice cysteine PI or cowpea serine PI can reduce *Rotylenchulus reniformis* female fecundity and density [9]. Constitutive expression of rice cysteine proteinase oryzacystatin I was effective against *Tobacco etch virus* (TEV) and *Potato virus Y* (PVY) in transgenic *N. benthamiana* plants [10].

*Cucumis metuliferus* (horned melon) is native to South Africa, is a highly nutritious source and is also reported to have resistance against many pathogens [11]. *C. metuliferus* line PI 292190 is immune to *Papaya ringspot virus* (PRSV), a member of the genus *Potyvirus* of the family *Potyviridae*, but *C. metuliferus* line Acc. 2459 is susceptible [12]. *C. metuliferus* line PI 292190 has a single dominant resistant gene, *Wmv*, against PRSV and has been used for selecting attenuated strains of PRSV [13]. This has made *C. metuliferus* an ideal model in studying plant-virus interaction and pathogenicity of PRSV in plants. In a previous study, we identified several transcript derived fragments (TDFs) from PI 292190 using cDNA-amplified fragment length polymorphism (cDNA-AFLP) [14]. One of these TDFs showed sequence similarity to serine proteinase inhibitor genes. This TDF was induced at 48 hour post inoculation in PRSV-resistant line PI 292190 but much later in PRSV-susceptible line Acc. 2459 (21 dpi) after PRSV infection. To test the function of *C. metuliferus* serine proteinase inhibitor (*CmSPI*), RNAi (RNA interference) was conducted in this study. RNAi initiated by double stranded RNA (dsRNA) modulates gene expression in eukaryotes via small interference RNA (siRNA) and microRNA (miRNA) [15,16]. RNAi is a powerful tool to study gene expression and analyze gene function [17]. The dsRNA can be formed *in vivo* by transforming plants with a construct that encodes hairpin RNA (hpRNA). Hairpin RNAi (hpRNAi) approach can lead to off target effects such as down-regulation of endogenous genes sharing sequence similarity with the hpRNAi construct. Moreover, an intron or inter-space contained self-complementary hpRNA (ihpRNA) construct could be more effective in its silencing ability [18].

This study has successfully obtained *CmSPI* RNAi transgenic lines in the PI 292190 genetic background, which showed a down regulation of *CmSPI* and suppression of the anti-PRSV resistance in *C. metuliferus* line PI 292190. However, transformation on the PRSV-susceptible *C. metuliferus* line Acc. 2459 was shown to be difficult. Therefore a full length genomic *CmSPI* fragment was cloned and used to transform *N. benthamiana*. Since PRSV could not infect *N. benthamiana*, the testing for resistance in transgenic *N. benthamiana* plants was conducted using a related potyvirus, *potato virus Y* (PVY), which is capable of infecting *N. benthamiana*. Two of the *N. benthamiana* transgenic lines were shown to be resistant to PVY infection. This study has provided evidence that the function of *C. metuliferus* *CmSPI*, a serine proteinase inhibitor gene, plays an important role in potyvirus resistance in both *C. metuliferus* and *N. benthamiana*.

## 2. Materials and Methods

### 2.1. Cloning CmSPI Gene cDNA and Full Length Genomic Fragment of Cucumis metuliferus

The *C. metuliferus* (PI 292190) cDNA fragment was identified using CapFishing Full-length cDNA Premix Kit (Seegene, Inc., Seoul, Korea) and the full length genomic fragment was identified by cassette ligation-mediated PCR genome walking [19]. In order to synthesize *CmSPI* cDNA, total RNA was isolated using previously described methods [20]. Three micrograms of RNA extracted from *C. metuliferus* line PI 292190 at 48 h post PRSV inoculation (hpi) were used for first strand cDNA synthesis, and the complementary strand was produced using the oligo-dT adaptor primer and the 5′ end of cDNA was ligated with CapFishing adaptor using reverse transcriptase. The mixture was then used for 3′ and 5′ rapid amplification of cDNA end (RACE) PCR reaction using specific primers HMK2007-14 and HMK2007-15 (Table 1). The PCR products were ligated into the cloning vector yT & A (Yeastern Biotech Co., Taipei, Taiwan) for sequencing. To identify full length *CmSPI* genomic fragment, 5 μg of DNA extracted from *C. metuliferus* line PI 292190 were digested with restriction enzymes (*Bam*HI, *Pst*I, *Eco*RI, *Eco*RV, *Hpa*I, *Nco*I, *Bst*XI, *Hin*dIII, *Kpa*I, *Sal*I, and *Xba*I) and then ligated with adaptors HMK2010-35 and HMK2010-36. PCR primers (HMK2010-37, HMK2010-38, HMK2011-118, HMK2011-119, HMK2011-127, and HMK2011-128) were used for genome walking (shown in Table 1) and the PCR products were then ligated into cloning vector yT & A for sequencing.

**Table 1:** Specific primers used in this study.

Primer Name	Oligonucleotides
**5′ and 3′ RACE reaction**	
HMK2007-14	5′-AAT TCC AAC ACA AAT CAT CAT CTT-3′
HMK2007-15	5′-TAA CAA ACA ACC AAA CTC GAT CAC-3′
**Full length genome walking**	
HMK2010-35	5′-CTA ATA CgA CTC ACT ATA ggg CTC gAg Cgg CCg CCC ggg CAg gT-3′
HMK2010-36	5′-ACC TgC CC-3′
HMK2010-37	5′-ggA TCC TAA TAC gAC TCA CTA TAg ggC-3′
HMK2010-38	5′-ACT CAC TAT Agg gCT CgA gCg ggC-3′
HMK2011-118	5′-TAT TCC AAC AAG TTC CGG CCA CTG-3′
HMK2011-119	5′-GAA AGT TGT TGA AAT TCC GAA GGT TG-3′
HMK2011-127	5′-gct gga act gga acc act aaa ga-3′
HMK2011-128	5′-cca taa atg gaa aca tac cag gag-3′
**CmSPI RNAi construction**	
HMK2007-93	5′-ggA TCC CCA Tgg CCC ggg CgA ATT CCA AgC TT-3′
HMK2007-94	5′-TgT ACA CTC gAg TAg Agg ggA TCC AgA TCT-3′
**Full length genomic CmSPI construction**	
HMK2012-6	5′-CAA ATA GGA GAA GAT GTT CTC G-3′
HMK2012-23	5′-CAG GTG ACA CAT GCG TAT AAC-3′
**Genomic PCR and probe synthesis**	
HMK2011-102	5′-gaa ctt tct gga tct act tta ttt g-3′
HMK2011-103	5′-aca caa act tca tct aac ctt aaa c-3′
**RT-PCR and probe synthesis**	
HMK2013-60	5′-acg cGA ATT CGT CGA CCT CGA GAA CCT TCG GAA TTT CAA CAA C-3′
HMK2013-61	5′-acg cGG ATC CGA GCT CAA GCT TAT GGC TGA TAT TTG TCC TCC T-3′

### 2.2. Sequence Analysis and Construction of Phylogenetic Tree

*CmSPI* sequence and the reactive sites were analyzed in the plant cis-acting regulatory DNA elements database [21]. The *CmSPI* ORF (KR012492) was translated into an amino acid sequence and used in phylogenetic analysis. The sequences of fifteen other serine proteinase inhibitor proteins were obtained including *Solanum tuberosum* (CAA78259, AAZ08247, ACZ04396), *Solanum lycopersicum* (AAA34198, AAA60745), *Nicotiana tabacum* (CAA78269), *Nicotiana sylvestris* (AAA34067), *Arabidopsis lyrata* (EFH39906), *Vitis cinerea* (ADD51184), *Ricinus communis* (EEF41422), *Jatropha curcas* (ADB85100), *Salvia miltiorrhiza* (ABP01767), *Medicago truncatula* (AES61046), and *Populus trichocarpa* (EEF01895) by BLASTP algorithm in the NCBI GenBank. These sequences were aligned using CLUSTAL W software (EMBL Data Library, Heidelberg, Germany), and a dendrogram was constructed with MAGA2 [22] using the neighbor-joining method [23] with the HKY85 [24] genetic distance. Data were resampled 100 times for bootstrap analyses.

### 2.3. Construction of Binary Vector and Bacterial Strain

The cDNA fragment of *CmSPI* gene for the RNAi construct was amplified by PCR with the primers, HMK2007-93 and HMK2007-94 (Table 1), and ligated into the yT & A vector. Sense and antisense *CmSPI* sequences were digested with *BamH* I–*Nco* I and *BsrG* I–*Xho* I individually and ligated into the pEPJ86i plasmid vector [25], in which the sense and antisense fragments were located in tandem with an inter-space sequence between them, and this ihpRNA construct was placed behind the cauliflower mosaic virus (CaMV) 35S promoter. The entire RNAi construct was then subcloned into the binary vector pGA482G and introduced into *Agrobacterium tumefaciens* strain LBA4404 for *Agrobacterium*-mediated transformation in *C. metuliferus*.

The full length genomic sequence of *CmSPI* gene was amplified from *C. metuliferus* resistant line PI 292190 with the primers, HMK2012-6 and HMK2012-23 (Table 1). The PCR reaction was conducted using the proof-reading DNA polymerase, Pfx DNA polymerase (Invitrogen^™^, Carlsbad, CA, USA). The resulting PCR product was digested with *Kpn* I and *Hpa* I and ligated into the binary vector pGA482G and introduced into *Agrobacterium tumefaciens* strain LBA4404 for *Agrobacterium*-mediated transformation into *N. benthamiana* plants.

### 2.4. Plant Material and Transformation

Transformation of *C. metuliferus* resistant line PI 292190 was conducted according to a previously described protocol [26]. In brief, cotyledon explants of the resistant line PI 292190 were infected with *A. tumefaciens* strain LBA4404 harboring the *CmSPI* RNAi binary vector construct. The resulting calli were screened for kanamycin resistance and the regenerated T_0_ plants were grown in soil-vermiculite mixture in pots. These T_0_ transgenic plants were tested by Southern hybridization and then self-pollinated to harvest T_1_ seed for RNA and protein analysis and virus inoculation test.

The transformation protocol for *N. benthamiana* was performed as previously described [27] with minor modifications. Young leaves harvested from two-month-old *N. benthamiana* plants were infected with *A. tumefaciens* strain LBA4404 carrying the full length genomic *CmSPI* binary vector construct. The resulting calli were screened for kanamycin resistance and regenerated T_0_ plants were grown in soil-vermiculite mixture in pots to harvest T_1_ and T_2_ seed. The T_1_ and T_2_ transgenic *N. benthamiana* lines were used for the virus inoculation test.

### 2.5. Inoculation of Plant Viruses

PRSV was obtained from *C. metuliferus* susceptible line Acc. 2459 leaves after virus inoculated. PVY obtained from infected *N. benthamiana* leaves was inoculated to transgenic *N. benthamiana* lines. Virus inoculation was conducted using a 1:50 (*w*/*v*) dilution of the homogenized leaves infected with the virus in 10 mM phosphate buffer (0.033 M K_2_H_2_PO_4_, 0.067 M K_2_HPO_4_ at pH 7.0).

### 2.6. Gene Expression Detection

#### 2.6.1. Genomic PCR

Genomic DNA was isolated from leaves of the transgenic *C. metuliferus* and *N. benthamiana* plants by cetyltrimethyl ammonium bromide (CTAB) method as previously described [28]. The presence of the transgene was detected by PCR with primer set HMK2011-102/HMK2011-103 for the intron sequence and HMK2013-60/HMK2013-61 primers for the *CmSPI* gene. PCR analysis was performed in a reaction mixture containing 10 μM specific primer pairs, 1× *Taq* DNA polymerase reaction buffer (50 mM KCl, 10 mM Tris-HCl, pH 9.0, 0.1% Triton X-100 and 1.75 mM MgCl_2_), 1U *Taq* DNA polymerase and 2.5 mM dNTP in a final volume of 20 μL.

#### 2.6.2. Southern Hybridization

Ten micrograms of genomic DNA from transgenic plants were digested with *Nco* I to completion, subjected to 1% agarose gel electrophoresis and transferred onto a nylon membrane (Perkin Elmer Life Science, Waltham, MA, USA). To detect the *CmSPI* RNAi construct in transgenic *C. metuliferus*, the membrane was hybridized with α^32^P-labeled intron sequence probe (generated by PCR with primer set HMK2011-102/HMK2011-103), and for detection of the full length genomic *CmSPI* construct in transgenic *N. benthamiana*, the membrane was hybridized with α^32^P-labeled *CmSPI* gene probe (generated by PCR with primer set HMK2013-60/HMK2013-61).

#### 2.6.3. RT-PCR (Reverse Transcription PCR)

The reverse transcription reaction was used Superscript III (Invitrogen^TM^) with HMK2013-61 as a primer for cDNA synthesis. The PCR using the specific primer set HMK2013-60/HMK2013-61was used for *CmSPI* gene expression detection.

#### 2.6.4. Northern Hybridization

Ten micrograms of total RNA from *C. metuliferus* line PI 292190 infected with PRSV at 48 hpi were subjected to denaturing electrophoresis in a 1% agarose gel containing 5% MOPS and then transferred onto a nylon membrane. The membrane was hybridized with α^32^P-labeled *CmSPI* gene probe (generated by PCR with primer set HMK2013-60/HMK2013-61 for *CmSPI* gene expression detection.

#### 2.6.5. Western Hybridization

Total protein was extracted from transgenic plants subjected to 16% poly-acrylamide gel electrophoresis, and transferred onto PVDF blotting membrane (GE Healthcare, UK). Immunostaining used anti-CmSPI polyclonal antibodies (CmSPI anti rabbit) and the one-step BCIP/NBT detection system (Pierce, Rockford, IL, USA).

#### 2.6.6. Detection of Small Interfering RNA

Ten micrograms of total RNA extracted from PRSV-infected *C. metuliferus* line PI 292190 at 48 hpi were subjected to 15% poly-acrylamide gel electrophoresis, and transferred onto a nylon membrane. The membrane was hybridized with α^32^P-labeled *CmSPI* gene probe (generated by PCR with primer set HMK2013-60/HMK2013-61) to detect *CmSPI* gene expression.

## 3. Results

### 3.1. Cloning and Functional Characterization of C. metuliferus CmSPI Gene

**Figure 1:**
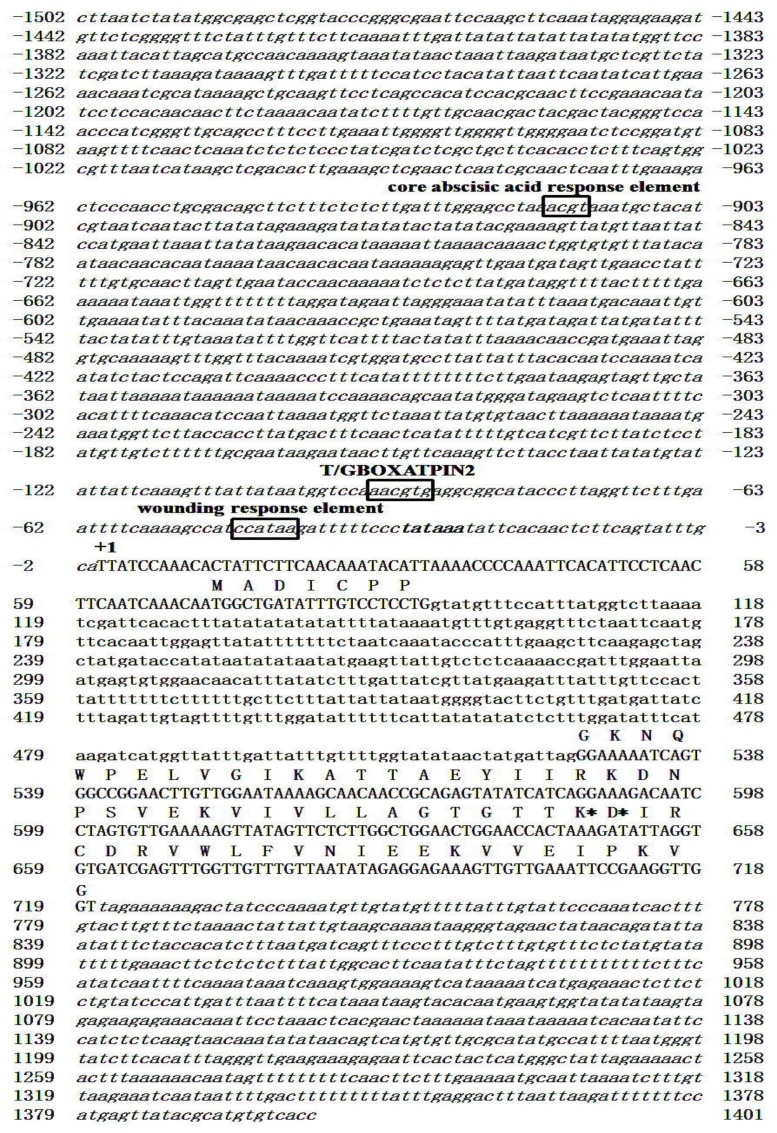
Nucleotide sequence of *CmSPI*. The nucleotide sequence of *C. metuliferus* *CmSPI* gene, including the 5′-flanking region and 3′-UTR, is shown. The transcription start site is labeled +1, and the TATA box is shown in boldface. The core abscisic acid response element, T/GBOXATPIN2 element, and wound response element are boxed. Asterisk marks indicate the predicted reactive sites, K48 and D49. Flanking region sequences are shown in italic letters. Exon and intron predicted sequences are shown in upper and lower case letters, respectively.

To identify full length cDNA and genomic fragments of *CmSPI*, RACE and genome walking were conducted. The ORF of *CmSPI* (KR012492) was identified as 219 bp in length, and the coding region of genomic fragment including 5′ and 3′ UTR was identified as 888 bp, containing two exons and one intron as shown in Figure 1. *CmSPI* sequence was analyzed in plant cis-acting regulatory DNA elements database, and the predicted reactive site of *CmSPI* was located at Lys48 (K48) and Asp49 (D49).

In addition, several conserved sequences of cis elements were identified in *CmSPI* promoter or upstream region. The “ACGT” sequence, a core abscisic acid response element [29], was found at −917 bp on the promoter region. The T/GBOXATPIN2 element “AACGTG” (identified in *Arabidopis thaliana* [30]) was present at −89 to −94 of *CmSPI*. Another element “CCATAA” (−42 to −47), a wound response element, is present on the *nopaline synthae* promoter [31] (Figure 1).

**Figure 2:**
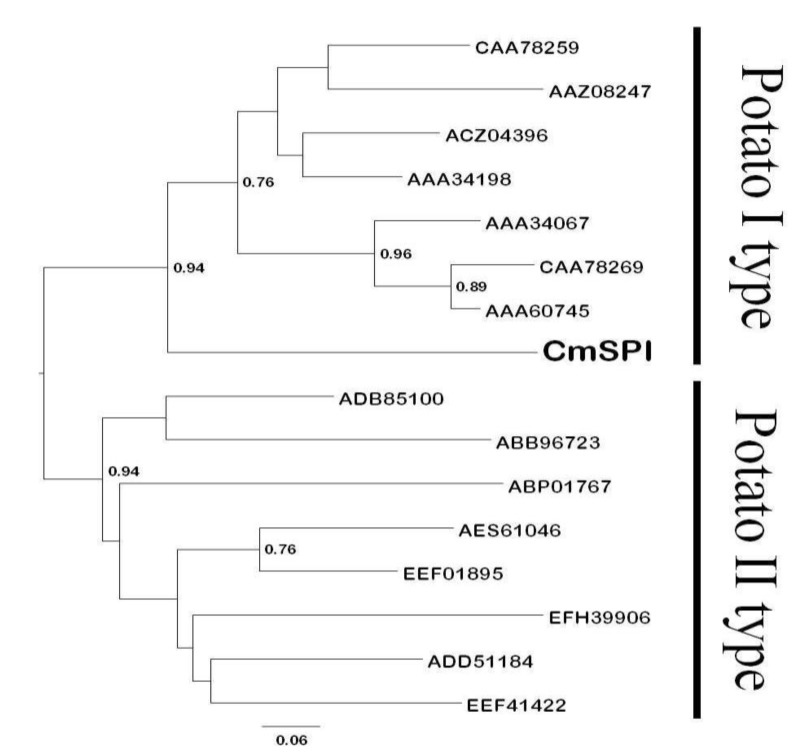
Neighbor-joining reconstruction of the phylogenetic relationships among plant serine proteinases inhibitors. Bootstrap values for nodes supported in >70% of 100 bootstrap replicates are shown above the branches. The scale bar shows the ratio of amino acid substitutions for a given horizontal branch length. The following reference sequences were included: *Solanum tuberosum* (CAA78259, AAZ08247, ACZ04396), *Solanum lycopersicum* (AAA34198, AAA60745), *Nicotiana tabacum* (CAA78269), *Nicotiana sylvestris* (AAA34067), *Arabidopsis lyrata* (EFH39906), *Vitis cinerea* (ADD51184), *Ricinus communis* (EEF41422), *Jatropha curcas* (ADB85100), *Salvia miltiorrhiza* (ABP01767), *Medicago truncatula* (AES61046), and *Populus trichocarpa* (EEF01895).

Phylogenetic analysis of amino acid sequence data from plant serine type PIs was conducted by neighbor-joining methods with the HKY85 genetic distance model as shown in Figure 2. Two distinct subtypes, potato I type and potato II type, were identified and CmSPI was grouped into potato I type which is a small size protein (8 kDa) containing two reactive sites.

### 3.2. Generation of CmSPI RNAi Transgenic Plant

**Figure 3:**
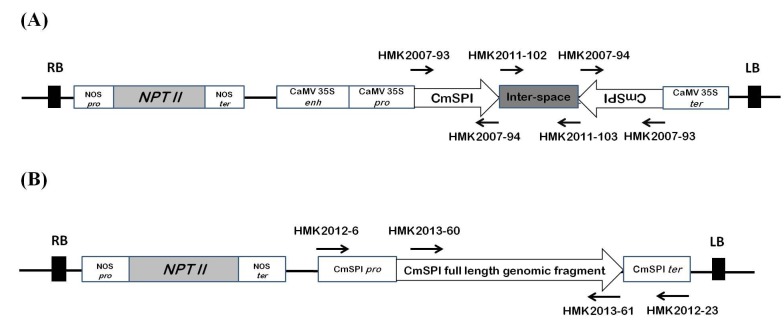
Constructs used for *C. metuliferus* and *N. benthamiana* transformation. (**A**) Construct for *C. metuliferus* *CmSPI* RNAi transformation. Partial sequence of *CmSPI* (203bp; from +46 to +692 nucleotide without intron sequences) was constructed into an invert-repeat form; (**B**) The construct for the transformation of full length genomic *CmSPI* into *N. benthamiana* plants. Arrows indicate the location of the primers used in the construction. RB, right border; Nos *pro*, nopaline synthase promoter; *NPT II*, neomycin phosphotransferase II; NOS *ter*, nopaline synthase terminator; CaMV 35S *enh*, cauliflower mosaic virus 35S enhancer; CaMV 35S *pro*, cauliflower mosaic virus 35S promoter; CaMV 35S *ter*, cauliflower mosaic virus 35S terminator; LB, left border.

**Figure 4:**
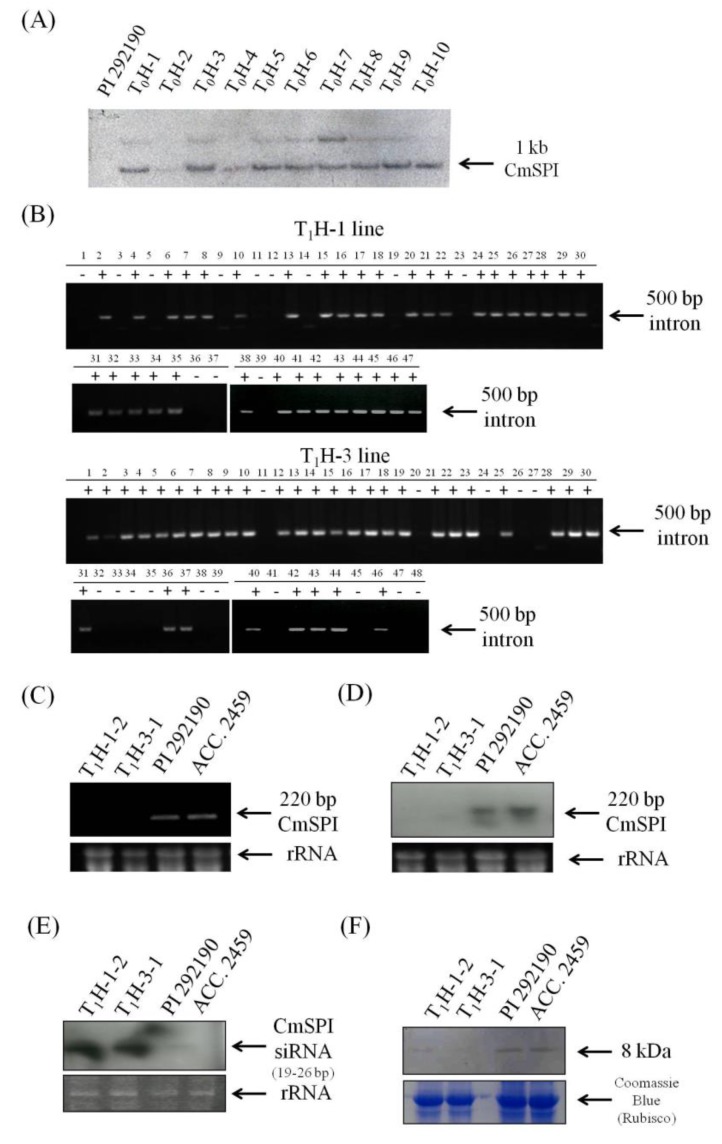
Generation of *CmSPI* RNAi trangsenic *C. metuliferus*. (**A**) Southern hybridization analysis of DNA isolated from RNAi T_0_ transgenic lines (T_0_H-1 to T_0_H-10) with α^32^P-labeled intron fragment probe. *C. metuliferus* line PI 292190 was used as a negative control; (**B**) PCR analysis of two RNAi transgenic T_1_ lines (T_1_H-1 and T_1_H-3 lines) with intron specific primers. +, transgenic plants; –, non-transgenic plants; (**C**) Transcript levels of *CmSPI* in *C. metuliferus* lines (PRSV-resistant line PI 292190 and PRSV-susceptible line Acc. 2459) and two T_1_ transgenic lines (T_1_H-1-2 and T_1_H-3-1). Total RNA from PRSV inoculated plants at 48 hpi was analyzed by RT-PCR. The ethidum bromide-stained rRNA is shown as a loading control; (**D**) Northern hybridization analysis of *CmSPI* in *C. metuliferus* lines (PI 292190 and Acc. 2459) and two T_1_ transgenic plants (T_1_H-1-2 and T_1_H-3-1). Total RNA isolated from PRSV inoculated plants at 48 hpi and detected with α^32^P-labeled *CmSPI* probe. The ethidum bromide-stained rRNA is shown as a loading control; (**E**) Detection of short interfering RNAs in *C. metuliferus* lines (PI 292190 and Acc. 2459) and two T_1_ transgenic plants (T_1_H-1-2 and T_1_H-3-1). Total RNA isolated from PRSV inoculated plants at 48 hpi and detected with α^32^P-labeled *CmSPI* probe. The ethidum bromide-stained rRNA is shown as a loading control; (**F**) CmSPI protein expression level in *C. metuliferus* lines (PI 292190 and Acc. 2459) and transgenic *CmSPI* RNAi plants (T_1_H-1-2 and T_1_H-3-1) by Western hybridization. Total soluble extracts from PRSV inoculated plants at 48 hpi and immunostaining with an anti-CmSPI polyclonal antibodies (CmSPI anti rabbit). The commassie blue-stained Rubisco is shown as a loading control.

To study *CmSPI* gene function by reverse genetics, an RNAi construct was generated for ihpRNA, constitutively expressing an inverted repeat of 203 bp *CmSPI* fragment (from +46 to +682 nt except intron sequence) under the control of the cauliflower mosaic virus (CaMV) 35S promoter. *C. metuliferus* PRSV-resistant line PI292190 was transformed by *Agrobacterium*-mediated transformation with the *CmSPI* ihpRNA construct (Figure 3). Southern hybridization (Figure 4A) confirmed that eight out of ten kanamcyin resistant plants (T_0_ generation) included the transgene and harbored the same size, indicating that these transgenic plants might be generated from the same calli. Two of these transgenic plants, T_0_H-1 and T_0_H-3, were self-pollinated to generate T_1_ seeds and the T_1_ progeny were then screened by genomic PCR (Figure 4B). Both T_1_H-1 line (47 plants, χ^2^ = 0.211, *p* = 0.487) and T_1_H-3 line (48 plants, χ^2^ = 1.778, *p* value = 0.317) showed goodness of fit to a 3:1 ratio, a segregation pattern indicative of a single dominant transgene inheritance in both cases. The resistant control line PI292190, susceptible control line Acc. 2459, and two T_1_ transgenic lines (T_1_H-1-2 and T_1_H-3-1) were then analyzed by RT-PCR, Northern hybridization for detecting high molecular weight or low molecular weight (small) RNAs, and Western hybridization. A decreased expression of *CmSPI* transcripts relative to the PRSV-resistant line PI292190 was evident (Figure 4C,D). For small interfering RNA (siRNA) analysis, the abundant accumulation of low molecular weight siRNA in the transgenic lines, a hallmark of RNA silencing, was detected (Figure 4E). To examine protein level in the RNAi transgenic lines, Western hybridization was conducted with a polyclonal antibody against CmSPI. A clear band (8 kDa) was detected in individual wild type controls (PRSV-resistant line PI 292190 and PRSV-susceptible line Acc. 2459) but a very faint band was shown in each individual transgenic lines (T_1_H-1-2 and T_1_H-3-1) (Figure 4F). In summary, both RNA and protein levels of *CmSPI* expression were down regulated greatly by the silencing effect of *CmSPI* ihpRNA construct.

### 3.3. Alteration of Virus Resistance in CmSPI RNAi Transgenic C. metuliferus

**Figure 5:**
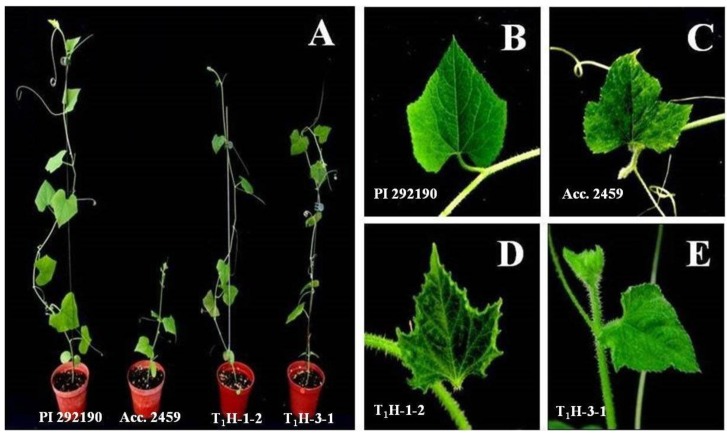
Effect of *CmSPI* gene silencing on PRSV inoculated *C. metuliferus* plants. (**A**) PRSV symptoms on the whole plant are shown for *C. metuliferus* reistance line PI 292190, susceptible line Acc. 2459, *CmSPI* RNAi transgenic line T_1_H-1-2, and *CmSPI* RNAi transgenic line T_1_H-3-1 (from left to right); PRSV symptoms on leaves for (**B**) PI 292190; (**C**) Acc. 2459; (**D**) transgenic line T_1_H-1-2; and (**E**) transgenic line T_1_H-3-1.

To examine the effect of silencing *CmSPI* in PRSV-resistance *C. metuliferus*, a total of 95 T_1_ transgenic plants (47 T_1_H-1 and 48 T_1_H-3 plants) along with three *C. metuliferus* resistant control plants (PI292190), and three *C. metuliferus* susceptible control plants (Acc. 2459) were inoculated with PRSV. The susceptible control Acc. 2459 showed typical symptoms of PRSV infection at 7 day post inoculation (dpi), the development of prominent mosaicism and chlorosis of leaves, water soaked streaking on the petiole and upper stem, and the distortion of young leaves (Figure 5A,C). The resistant control PI292190 showed immunity against PRSV and no symptoms were detected (Figure 5A,B). After silencing *CmSPI* on the resistant line PI292190, *CmSPI* RNAi T_1_ transgenic plants were losing immunity to PRSV and showed PRSV infection symptoms on the topmost leaves after PRSV inoculation at 21 dpi (Figure 5D,E). This indicated that silencing of *CmSPI* gene could break down the resistance to PRSV in these RNAi transgenic lines.

### 3.4. Resistance to Virus in Transgenic N. benthamiana

**Figure 6:**
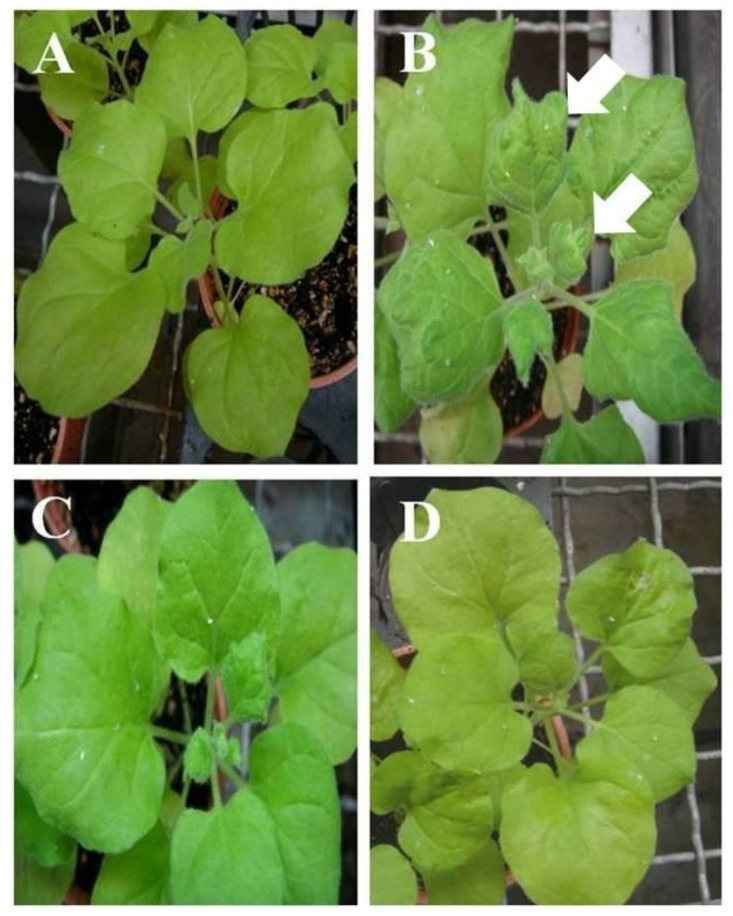
PVY resistance of *CmSPI* T_1_ transgenic *N. benthamiana* plants at 14 dpi. (**A**) Wild type *N. benthamiana*-mock; (**B**) Wild type *N. benthamiana*-PVY inoculated; (**C**) T_1_F-21-2- PVY inoculated; (**D**) T_1_F-51-10- PVY inoculated. Arrow indicates the symptoms of PVY on *N. benthamiana*.

**Figure 7:**
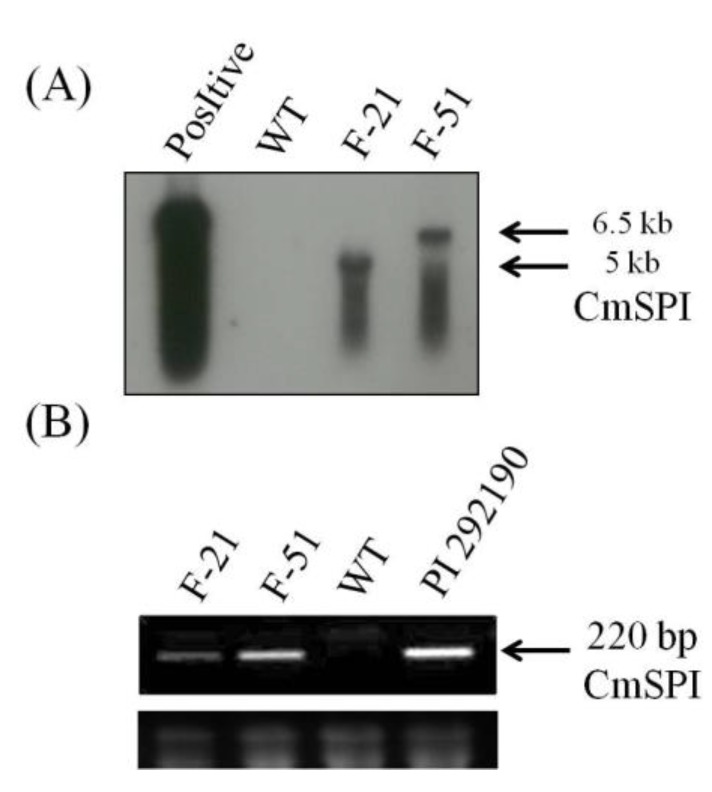
Southern hybridization and RT-PCR analysis of *CmSPI* T_2_ transgenic *N. benthamiana* lines. (**A**) Southern hybridization analysis of DNA isolated from wild type *N. benthamiana* control (WT), *CmSPI* T_2_ transgenic lines, T_2_F-21-2-5 and T_2_F-51-10-8, with α^32^P-labeled *CmSPI* probe. *CmSPI* transformation plasmid DNA was used as a postive control; (**B**) Transcript levels of *CmSPI* in postive control (*C. metuliferus* resistance line PI 292190), negative control WT (wild type *N. benthamiana* plant), and *CmSPI* T_2_ transgenic lines (T_2_F-21-2-5 and T_2_F-51-10-8). Total RNA from PVY inoculated plants at 2 dpi was analyzed by RT-PCR. The ethidum bromide-stained rRNA is shown as a loading control.

To confirm the function of *CmSPI* in potyviruses resistance, the full length *CmSPI* genomic fragment with size 2858 bp (from −1457 to +1411 nt) was cloned and transformed into *N. benthamiana* by *Agrobacterium*-mediated transformation. All transgenic plants showed no obvious morphological alterations as compared to wild type *N. benthamiana*. Eleven T_0_ transgenic plants were tested by PCR analysis and nine of them showed *CmSPI* gene positive signal (data not shown). The T_0_ lines with transgenes were selected and self-pollinated to harvest seeds. T_1_ plants derived from individual T_0_ transgenic lines along with wild type *N. benthamiana* control plants were inoculated with PVY. As shown in Table 2, two of these transgenic lines, T_1_F-21 and T_1_F-51, showed some degree of PVY resistance. The symptoms of PVY infection were observed on those non-transformed *N. benthamiana* plants at 7 dpi. Compared to wild type *N. benthamiana* control plants, these two lines showed no symptoms at 14 dpi (Figure 6). Two lines (T_1_F-21-2 and T_1_F-51-10) selected from the T_1_ population were then self-pollinated to generated T_2_ plants. Using Southern blot, two T_2_ lines, T_2_F-21-2-5 and T_2_F-51-10-8, showed one copy of the *CmSPI* insertion (Figure 7A). In RNA level, *CmSPI* gene expression was observed after virus inoculation at 2 dpi in T_2_ transgenic plants in which RNA expression levels were lower than those in *C. metuliferus* control PI 29219 (Figure 7B). Furthermore, T2 plants generated from independent T_1_ lines (T_1_F-21-2 and T_1_F-51-10) and three wild type *N. benthamiana* control plants were inoculated with the sap obtained from wild type *N. benthamiana* plants infected with PVY. Different levels of viral resistance were observed in these two lines. A total of two out of 44 plants of T_2_F-51-10 line showed PVY infected symptoms, but 33/44 of T_2_F-21-2 plants showed PVY infected symptoms at 21 dpi (Table 3). This result indicated the resistant function of *CmSPI* expression against PVY was identified in *N. benthamiana* transgenic plants.

**Table 2:** Infectivity assays of T_1_ transgenic *N. benthamiana* plants inoculated with PVY.

Transgenic Lines	Symptomatic Plants/Total of Inoculated Plants
T_1_F-3	28/28
T_1_F-5	25/25
T_1_F-14	21/22
T_1_F-21	18/30
T_1_F-51	17/30
T_1_F-52	27/30
T_1_F-53	29/29
T_1_F-54	30/30
T_1_F-55	27/28
Non-transformed control	27/27

The values indicate the number of plants with symptoms of disease (left of slash) and total number of inoculated plants (right of slash). Plants were observed for up to 21 days post-inoculation.

**Table 3:** Infectivity assays of T_2_ transgenic *N. benthamiana* plants inoculated with PVY.

Transgenic Lines	Symptomatic Plants/Total of Inoculated Plants
T_2_F-21-2	33/44
T_2_F-51-10	2/44
Non-transformed control	15/15

The values indicate the number of plants with symptoms of disease (left of slash) and total number of inoculated plants (right of slash). Plants were observed for up to 21 days post-inoculation.

## 4. Discussion

### 4.1. CmSPI Is Involved in Plant Physiological Responses and Plant Defense

In our previous study, TDFs isolated from *C. metuliferus* were shown to be related to PRSV resistance using cDNA-AFLP analysis, and one of the candidate genes, *CmSPI*, was strongly induced after PRSV inoculation at 48 hpi [14]. In this study, we cloned and identified the full length sequence of the *CmSPI* gene from PRSV resistant line PI 292190. The ORF of *CmSPI* was identified as 219 bp in length and codes for a small protein (8 kDa) classified as a serine type proteinase inhibitor belonging to the serpin (serine PI) family, the largest and most widespread superfamily of PIs [32]. Plant PIs are active against different classes of proteinases and have been classified into different families [6]. They have been reported to interact with a variety of factors involving developmental and protein storage signals, wounding responses, and abiotic and biotic stress responses [33,34,35,36,37]. For example, peanut Bowman-Birk inhibitor genes, *AhBBI*, encode serine protease inhibitors that respond to drought stress, exogenous jasmonic acid (JA), and abscisic acid (ABA) [38]. The expression of soybean cysteine PI genes, *R1* and *N2*, was induced by wounding and methyl jasmonate treatments, indicating a role for *RI* and *N2* in plant defense [39]. The present study also detected several cis elements in the promoter region of the *C. metuliferus* *CmSPI* including a core abscisic acid response element [29], a T/GBOXATPIN2 element [30], and a wound response element indicating the *CmSPI* gene is involved in the responses to ABA, JA, and wounding [31].

### 4.2. Silencing of CmSPI Alters PRSV Resistance in C. metuliferus

To confirm the antiviral function of *CmSPI* in PRSV resistance, RNAi was performed using an inter-space or intron contained self-complementary hpRNA construct to enhance the silencing efficiency. In the present study, the immune response against PRSV in *C. metuliferus* resistant line PI 292190 was broken down after silencing *CmSPI* transgene expression in *CmSPI* RNAi transgenic plants. However, the mosaic symptoms on the upper leaves of transgenic lines were observed in a delayed fashion, after 21 dpi, compared to the severe symptoms at 7 dpi in PRSV susceptible control Acc. 2459. This could be explained by the fact that the expression of *CmSPI* was knocked down instead of completely knocked out. This virus has to replicate and accumulate enough to overcome the threshold of basic defense to display symptoms in *CmSPI* RNAi transgenic lines. Alternatively, P1/Hc-Pro of PRSV is a strong virus silencing suppressor by binding to *CmSPI*-derived siRNAs [40], which might reduce the RNAi efficiency of *CmSPI* RNAi transgenic plants.

### 4.3. Expression of CmSPI in N. benthamiana Plants Can Supply Resistance for PVY

Plant viruses such as potyviruses, tymoviruses, nepoviruses, comoviruses, and closteroviruses would require proteinases to process their own proteins for replication and propagation [41]. Hence plant PIs might play a role in limiting proteinase function in these viruses. In a previous report, constitutive expression of rice cysteine PIs (*oryzacytatin I*) in transgenic *N. benthamiana* lines induced resistance against both *Tobacco etch virus* (TEV) and PVY infections [10]. Interestingly, a serine PI instead of cysteine PIs, CmSPI, identified in this study, was also shown to provide resistance function in *N. benthamiana* against PVY, a member of the potyvirus group. Potyviruses contains three proteinases (P1, HC-Pro, and NIa ) which have been reported in regulation of viral gene expression, replication and infection cycle [42,43]. It has been reported that a serine proteinase encoded by *P1* gene can self-cleave at its C terminus for its function and the reactive sites of P1 proteinase included Asp233, Gly-Ser-Ser256-Gly [43]. In this study, CmSPI is a serine type PI and its reactive site was located at the 48th (K48) and 49th (D49) amino acid residues which might obstruct the function of P1 serine proteinase of potyviruses. This suggests that plant PIs both cysteine or serine type potentially have a role in defending against different viruses. Interestingly, the T_2_ plants derived from two resistant T_1_ transgenic *N. benthamiana* lines showed variations (42/44 in T_2_F-51-10 and 11/44 in T_2_F-21-2) with regard to their resistance to PVY. It is not uncommon that variations in resistance occur among different generations of transgenic lines. For example, a previous study reported that R_1_ plants expressing N gene segments of *Tomato spotted wilt virus* (TSWV) showed variations in their resistance to viruses [44]. However, further experiments would be required to understand the association between the inheritance of the transgene to its target traits.

## 5. Conclusions

We have demonstrated that the *C. metuliferus* *CmSPI* gene encodes a serine proteinase inhibitor. Knocking down *CmSPI* gene expression in the PRSV-resistant *C. metuliferus* line PI 292190 broke down its resistance against PRSV. Furthermore, heterologous expression of the *C. metuliferus* *CmSPI* gene in *N. benthamiana* provided resistance to PVY, another *Potyvirus*, and indicated its important role in the mechanism of potyviruses resistance.
